# Implications of Continuous Noninvasive Finger Cuff Arterial Pressure Device Use during Cesarean Delivery for Goal-Directed Fluid Therapy Preload Optimization: A Randomized Controlled Trial

**DOI:** 10.1155/2021/6685584

**Published:** 2021-03-28

**Authors:** Shan-Han Yang, Yi-Shiuan Lin, Chien-Nan Lee, Ya-Jung Cheng, Ying-Hsi Chen, Hsin-Chan Chiu, Chun-Yu Wu

**Affiliations:** ^1^Department of Anesthesiology, National Taiwan University Hospital, No. 7, Chung-Shan S. Rd., Taipei, Taiwan; ^2^Department of Anesthesiology, Taiwan Adventist Hospital, No. 424, Sec. 2, Bade Rd., Songshan District, Taipei, Taiwan; ^3^Department of Obstetrics and Gynecology, National Taiwan University Hospital, No. 7, Chung-Shan S. Rd., Taipei, Taiwan

## Abstract

**Background:**

Although fixed-volume conventional fluid preloading protocol fails to attenuate postspinal hypotension during cesarean delivery, the effect of goal-directed fluid therapy (GDFT) remains less explored. Continuous noninvasive finger cuff arterial pressure monitoring using devices such as the ClearSight System can provide the noninvasive stroke volume value, enabling clinicians to perform GDFT before spinal anesthesia; however, the efficacy of GDFT requires further elucidation.

**Method:**

In total, 71 consecutive full-term pregnant women were randomly divided into a control group (*n* = 34) and a GDFT group (*n* = 37). Before spinal anesthesia, the control group received a fixed dose (1000 mL) of crystalloid fluid, but the GDFT group received repeated 3 mL/kg body weight of crystalloid fluid challenges within 3 minutes with a 1-minute interval between each fluid challenge based on the stroke volume incremental changes obtained using the ClearSight System (targeting a stroke volume increase of ≥5% after a fluid challenge). The primary outcome was the incidence of postspinal hypotension. The secondary outcomes were total fluid volume, vasopressor dosage, hemodynamic parameter changes, maternal adverse effects, and neonatal profiles.

**Result:**

Women in the GDFT group received more fluid than did those in the control group (1132 ± 108 vs. 1247 ± 202 mL; *p* = 0.0044), but the incidence of postspinal hypotension (79.4% vs. 73.0%,; *p* = 0.5864) and norepinephrine dose (12.5 ± 10.6 vs. 15.1 ± 12.8 mcg, respectively; *p* = 0.3512) was comparable between the two groups. Fewer women in the GDFT group experienced nausea (61.76% vs. 35.14%; *p* = 0.0332). Neonatal outcomes (Apgar score and umbilical blood analysis) were comparable and typical in both groups.

**Conclusion:**

ClearSight-guided GDFT did not ameliorate postspinal hypotension but may reduce nausea. This trial is registered with NCT03013140.

## 1. Introduction

Postspinal hypotension, defined as a 20% reduction in baseline systolic blood pressure (SBP), is prevalent during cesarean delivery [1] and is correlated with maternal adverse effects, such as nausea, as well as neonatal profiles. Intraoperative hypotension is often treated using fluid therapy [2], but it only provides limited efficacy during cesarean delivery [1, 3]. This may be because goal-directed fluid therapy (GDFT) is the most efficient form of intravenous fluid therapy [2], but previous literature has mostly emphasized fixed-volume fluid administration [1]. This may be because the GDFT protocol mandates the use of a continuous cardiac output monitoring system, but monitoring systems such as esophageal Doppler monitoring or arterial pressure pulse contour analysis are too invasive for women undergoing cesarean delivery.

The ClearSight System (Edwards Lifesciences, Irvine, CA, USA) can noninvasively calculate the continuous stroke volume and cardiac output via the volume-clamp method with an internal physiologic calibration procedure [4]. This allows clinicians to noninvasively initiate the GDFT protocol in women undergoing cesarean delivery. Therefore, this study investigated the effects of the GDFT fluid preloading protocol on the amelioration of postspinal hypotension and maternal adverse effects.

## 2. Materials and Method

### 2.1. Participants and Group Allocation

Ethical approval for this study (201610025RIND) was provided by the Research Ethics Committee of National Taiwan University Hospital, Taipei, Taiwan. After written informed consent was obtained from all parturients on the day before surgery, women undergoing elective cesarean delivery were enrolled consecutively from March 2017 to November 2018 (NCT03013140). We excluded those who received emergent cesarean delivery, received failed spinal sensory targeted level (T6), were obese (body mass index > 35 kg/m^2^), had a height < 150 cm or >175 cm, had a gestational age < 36 weeks, and had pregnancy-induced hypertension, preeclampsia, or multiple pregnancies.

Because of insufficient time to apply GDFT during spinal anesthesia (the coloading protocol), we conducted a GDFT-based preloading protocol (GDFT group) to compare with the conventional fixed-volume fluid preload protocol (control group). On arrival at the operating theater, women were allocated to the study arms in a 1 : 1 ratio according to a computer-generated randomization list. Then, patients were divided into two groups: the control group and the GDFT group.

### 2.2. Hemodynamic Monitoring and Fluid Protocols

The allocation concealment was performed. The study participants and the attending anesthesiologist were blinded to the group allocation. After arrival to the operating room, each woman received standard monitoring including electrocardiographic monitoring, pulse oximetry, and oscillometric blood pressure measurement (IntelliVue MP70; Philips Electronics, Suresnes, France) and the ClearSight™ system. Before implementation of the fluid protocol, women rested in the supine position for at least 5 minutes in a quiet environment to obtain the appropriate hemodynamic state. Then, either the fixed-volume preloading or GDFT protocol was initiated before spinal anesthesia by an independent anesthesiologist who did not participate in anesthetic care. Women in the control group received a rapid infusion of Ringer's solution (1000 mL) within 15 minutes. The ClearSight™ system was monitored in women in the control group, but the information was not revealed to the anesthesiologist. Women in the GDFT group received an individualized fluid therapy protocol which involved infusing Ringer's solution (3 mL/kg) within 3 minutes with a 1-minute interval between each fluid bolus for observation of stroke volume fluid responsiveness. A positive fluid response was defined as a stroke volume increase of >5% after fluid challenge [5–7]. Repeat fluid bolus was administered until the stroke volume was fluid unresponsive (<5% change) [8]. The data derived from the ClearSight System were recorded but were not accessible to the attending anesthesiologist in charge of anesthetic care.

The baseline blood pressure was measured after completion of the fluid protocol by using a continuous oscillometric sphygmomanometer in the arm in the supine position by calculating the mean of three consecutive readings [1]. After the patient had rested in the supine position for at least 5 minutes for hemodynamic stabilization, an epidural needle was inserted at the T11–12 vertebral interspace using the loss-of-resistance-to-air technique, and a multiorifice epidural catheter (PORTEX, Epidural Minipack, Smiths Medical, Czech Republic) was threaded 4–6 cm into the epidural space. An epidural test dose was not administered. Spinal anesthesia was administered by using a 27-G Quincke pencil point needle (Becton Dickinson; Oxford, UK) at L3–4 or L4–5 vertebral interspace with the woman in the left lateral decubitus position. After ensuring free flow of cerebrospinal fluid, the anesthesiologist administered 0.5% hyperbaric bupivacaine 10–12 mg and fentanyl 10 mcg over 30 seconds to achieve a cold and touch sensory blockade at the T6 level [9, 10]. Left uterine displacement was performed in all women before skin incision. Failed spinal anesthesia or inadequate sensory block for surgery requiring a rescue epidural dose or conversion to general anesthesia resulted in exclusion from the trial.

### 2.3. Management of Postspinal Hypotension

In both groups, treatment for hypotension was based on oscillometric BP values which were measured at 2.5-minute cycles during surgery. Postspinal hypotension was defined as <80% of baseline SBP [1]. Hypotension and adverse effects (e.g., nausea) following fetal delivery may be frequently induced by uterotonic agents [11, 12], and thus, the present investigation focused on the period before delivery to avoid bias in the observation of postspinal hypotension and maternal adverse effects. The hypotension was treated with intermittent bolus injection of intravenous norepinephrine 5 *μ*g per dose. If hypotension persisted after two doses of intravenous norepinephrine injection (10 *μ*g), 100 mL of Ringer's solution was rapidly coinfused with an additional norepinephrine dose. For hypotension with an SBP reduction > 30% of baseline, intravenous norepinephrine 10 *μ*g with 100 mL Ringer's solution rapid infusion was administered. For correction of bradycardia with a heart rate less than 60 beats per minute, intravenous atropine 0.5 mg was injected.

### 2.4. Parameters and Study Endpoints

Maternal hemodynamic parameters, including SBP (both oscillometric and ClearSight), heart rate, and stroke volume, and cardiac output were recorded before implementation of the fluid protocol, in the prespinal state (baseline; after fluid protocol and before spinal anesthesia), and every 2.5 minutes following spinal anesthesia until neonatal delivery. Because the median interval between spinal anesthesia and neonatal delivery was approximately 15–18 minutes based on our institutional records, the hemodynamic changes were specifically compared between the two groups at eight time points, namely, *T*_0_ (before fluid protocol), *T*_1_ (baseline; after fluid protocol and before spinal anesthesia), and at the following times after spinal anesthesia: *T*_2_ (2.5 minutes), *T*_3_ (5.0 minutes), *T*_4_ (7.5 minutes), *T*_5_ (10.0 minutes), *T*_6_ (12.5 minutes), and *T*_7_ (15.0 minutes).

Prior to neonatal delivery, the incidence of postspinal hypotension and maternal adverse effects including nausea, dizziness, dyspnea, bradycardia, and shivering and norepinephrine doses were recorded. After neonatal delivery, umbilical arterial (UA) and venous (UV) blood samples were collected from a double-clamped segment of umbilical cord and immediately measured using a blood gas analyzer (RAPIDPoint 500, Siemens Healthcare Diagnostics, USA). Apgar scores were evaluated at 1 and 5 minutes following neonatal delivery.

### 2.5. Statistical Analysis

The primary outcome is the incidence of postspinal hypotension. Based on the previous literature, the incidence of postspinal hypotension may be up to 70% [1, 13]. In addition, a previous study reported that GDFT may reduce the incidence of postspinal hypotension by 40% [14]. Thus, we estimated that a sample of 29 patients in each group was needed to obtain *α* (type I error) = 0.05 and *β* (type II error) = 0.1. A total of 40 patients were recruited in each group to compensate for the loss to follow-up and lack of research manpower at times because of potential emergency cesarean delivery at our institute. The secondary outcomes were total fluid volume, vasopressor dosage, hemodynamic parameter changes, maternal adverse effects, and neonatal profiles. A Fisher exact test or chi-squared test was employed to analyze dichotomous data, Student's *t*-test was used for normally distributed continuous data, and the Mann–Whitney *U* test was used for nonparametric ordinal data. Repeated measures analysis of variance with the group and time factors, followed by post hoc analysis with Tukey's test, was used to compare serially measured variables. Statistical analyses were performed using MedCalc (MedCalc, Mariakerke, Belgium).

## 3. Results

### 3.1. Participant Characteristics

**Figure 1:**
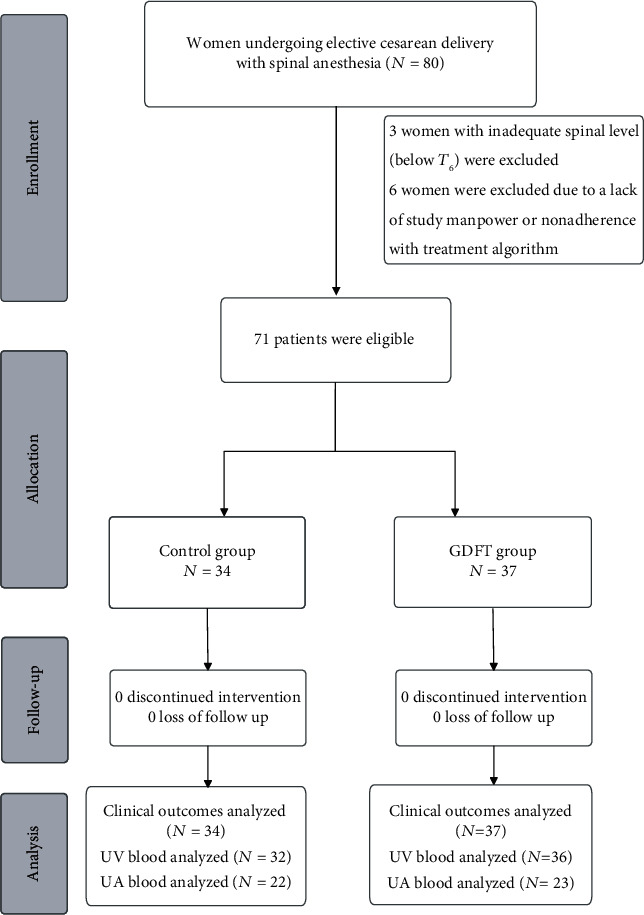
CONSORT flow diagram.

Figure 1 presents the CONSORT diagram of inclusion. A total of 80 women met the inclusion criteria and agreed to participate in this trial, and 9 women were excluded because of an inadequate spinal anesthesia level (sensory block below T6), lack of research manpower, and nonadherence to the treatment protocol. Women in the two groups presented comparable characteristics (Table 1).

### 3.2. Intraoperative Maternal Profiles

Table 2 summarizes the intraoperative maternal profiles. Women in the two study groups exhibited comparable intraoperative profiles except for women in the GDFT group who received more intravenous fluid than did those in the control group (1247 ± 202 vs. 1132 ± 108 mL; *p* = 0.0044). In the control and GDFT groups, the incidence of postspinal hypotension was comparably high (79.4% vs. 73.0%, respectively; *p* = 0.5864), and the norepinephrine dosage requirements were also comparable (12.5 ± 10.6 vs. 15.1 ± 12.8 *μ*g, respectively; *p* = 0.3512). Regarding spinal anesthesia–associated maternal adverse effects, fewer women in the GDFT group experienced nausea (61.76% vs. 35.14%, respectively; *p* = 0.0332). Women in both groups revealed comparable maternal adverse effects including the incidence of dizziness, bradycardia, and shivering (Table 2).

**Figure 2:**
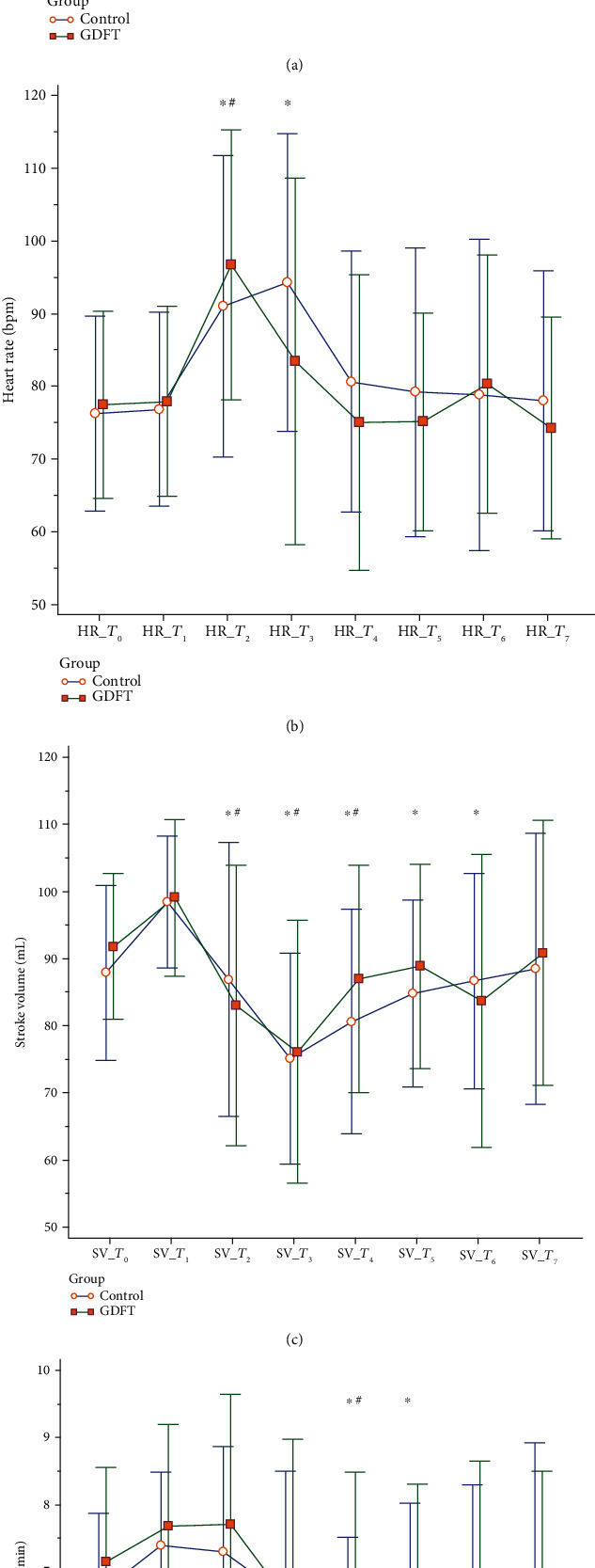
Hemodynamic changes prior to neonatal delivery: (a) oscillometric systolic blood pressure changes; (b) heart rate changes; (c) stroke volume changes; (d) cardiac output changes. ∗ denotes a significant difference compared with data at *T*_1_ in the control group. # denotes a significant difference compared with data at *T*_1_ in the GDFT group.

The oscillometric SBP values in both groups were significantly lower than the baseline values (*T*_1_) during *T*_3_–*T*_6_, but these values were maintained above 80% of the baseline values because of vasopressor treatment (Figure 2(a)). Spinal anesthesia induced significant heart rate elevations in both groups between *T*_1_ and *T*_2_. The heart rate value of women in the control group was significantly higher than the baseline value (*T*_1_) during *T*_2_–*T*_3_, whereas that of women in the GDFT group was significantly higher than baseline at *T*_2_ only. Both fluid protocols induced significant increases in stroke volume and cardiac output (Figures 2(c) and 2(d); between *T*_0_ and *T*_1_), and spinal anesthesia induced a significant reduction in stroke volume and cardiac output among women in both study groups (Figures 2(c) and 2(d); between *T*_1_ and *T*_2_). In comparison with the stroke volume values at prespinal states (*T*_1_), women in the GDFT group revealed a stroke volume reduction during the first 7.5 minutes after spinal anesthesia (*T*_2_–*T*_4_; Figure 2(c)). By comparison, women in the control group had more prolonged stroke volume reduction during the first 15 minutes after spinal anesthesia (*T*_2_–*T*_6_; Figure 2(c)). The cardiac output value changes were modest compared with changes in stroke volume after spinal anesthesia. In comparison with the cardiac output values at baseline (*T*_1_), women in the GDFT and control groups revealed a cardiac output reduction at *T*_5_ and at *T*_4_ and *T*_5_, respectively (Figure 2(d)). However, the differences in hemodynamic parameters between the two groups at each time point were nonsignificant.

### 3.3. Neonatal Profiles

The neonatal outcome is summarized in Table 3. The 1-minute and 5-minute Apgar scores were comparable between the groups. The 2 UV samples and the 12 UA samples in the control group and the 1 UV sample and the 14 UA samples in the GDFT group were insufficient for detection using the blood gas analyzer. Umbilical blood gas analysis results were comparable between the groups, except that women in the GDFT group exhibited a higher UA pCO_2_ (42.8 ± 5.1 vs. 46.4 ± 6.5 mmHg in the control and GDFT groups, respectively; *p* = 0.0465). However, values for all umbilical blood gas analyses were within normal range and without evidence of acidosis.

## 4. Discussion

The major findings of this study are as follows: First, a GDFT preload protocol was not more effective in ameliorating postspinal hypotension than a 1000 mL fixed-volume conventional preloading protocol; second, GDFT marginally improved postspinal stroke volume and reduced the incidence of postspinal nausea.

Despite the fixed-volume preload strategy being less effective in ameliorating postspinal hypotension, it was still recommended in the UK guidelines (2011) [15] and by the American Society of Anesthesiologists/Society for Obstetric Anesthesia and Perinatology Task Force 2016 [16]. A recent meta-analysis revealed that fixed-volume crystalloid preloading is the most commonly reported method to prevent postspinal hypotension during cesarean delivery among the 109 trials and 12 methods analyzed [17]. However, this meta-analysis indicated that fixed-volume crystalloid was not effective in ameliorating postspinal hypotension, with an odds ratio of 0.78 (0.46–1.31) [17]. The fluid volume used for the control group in this study (1000 mL of crystalloid) is the most common dose used in conventional fluid preloading protocol during cesarean delivery [17, 18]. Furthermore, the estimated EV_50_ of the preloaded crystalloid required to prevent spinal anesthesia-induced hypotension during cesarean delivery was reported 13 mL/kg body weight [19]. In the present study, the GDFT group received a mean volume of 1247 mL of crystalloid fluid (approximately 18 mL/kg body weight), which was higher than the previously reported EV_50_ [19]. Therefore, the GDFT protocol may optimize the stroke volume before spinal anesthesia; on the other hand, the women who received the fixed-volume fluid preloading protocol demonstrated a more prolonged stroke volume reduction after spinal anesthesia. However, our results revealed that the preloading fluid strategy in GDFT also did not improve postspinal hypotension during cesarean delivery. By comparison, Xiao et al. reported that GDFT may reduce hypotension incidence prior to delivery (hypotension incidence 62% vs. 20% in control and GDFT group, respectively) and obviated the need for vasopressors (no vasopressor used among women in the GDFT group) [14]. The current study is different from that of Xiao et al. in various respects. First, in the study by Xiao et al., women in both the control and GDFT groups received fluid coload. In addition, GDFT was initiated after the fluid coload, but women in the control group received no additional fluid treatment. This may result in bias during treatment comparison. Second, the GDFT protocol in the study of Xiao et al. was conducted during a hemodynamically unstable period (i.e., the interval between combined spinal epidural anesthesia and delivery). High variations in vascular tone due to spinal anesthesia and epidural top-up, positioning changes, autonomic reflexes, such as the Bezold-Jarisch reflex, and maternal adverse effects, such as nausea, may frequently occur and influence the hemodynamic changes during this short interval (typically shorter than 20 minutes). These factors may considerably impede the reliability of fluid responsiveness assessment for the GDFT protocol. Therefore, in the present study, we conducted the fluid protocol during the preload period before spinal anesthesia administration. During the preload period, a more stable condition could be achieved without bias to the GDFT protocol. The incidence of postspinal hypotension in the current study is consistent with the higher ranges reported in related studies [1]. This may be because of several reasons. First, no coloading fluid was administrated in this study to avoid bias when comparing the difference between GDFT preloading and conventional fixed-volume preloading fluid strategies. Second, a relatively high single-shot intrathecal bupivacaine dose was selected to achieve adequate sensory blockade without epidural supplementation. This dose was selected based on our institutional database, and it was close to the effective dose for 95% of single-shot spinal anesthesia [20, 21]. Consequently, only three of the participants (3.75%) exhibited a sensory blockade below *T*_6_. By comparison, most previous reports comprised a fixed intrathecal anesthetic dose, and the sensory dermatome was not targeted, which may cause bias.

We observed that GDFT may reduce intraoperative nausea incidence. The incidence of nausea observed in the current study (approximately 30%) was similar to that in a previous report with prophylactic norepinephrine infusion to prevent postspinal hypotension during cesarean delivery [22]. Despite GDFT not resulting in higher preanesthetic hemodynamic states, more iv fluid was administered, which could result in higher blood volume and more favorable stroke volume stabilization after spinal anesthesia. Because the gut perfusion is sensitive to blood volume and stroke volume changes [23] and spinal anesthesia could markedly reduce splanchnic blood flow [24], the benefit of additional iv fluid on the elevation of blood volume and stroke volume may be more prominent than its effects on hypotension amelioration. In addition, iv crystalloid may prevent the intraoperative elevation of antidiuretic hormone release, which results in the inhibition of the sensation of nausea [25]. This result is also compatible with studies in the general surgical population which indicated that supplemental intravenous crystalloid is associated with the amelioration of postoperative nausea [25]. However, continuous vasopressor infusion may remain more effective in preventing both postspinal hypotension and nausea [26, 27] than fluid infusion. In terms of its effects on postspinal hypotension and nausea prevention, vasopressors should still be considered the first-line treatment, but the GDFT may be a useful adjunct therapy.

The ClearSight System provides numerous hemodynamic parameters including stroke volume, cardiac output, and stroke volume variation. In this study, we assessed the effects of fluid challenge mainly based on stroke volume elevation because cardiac output may be affected by variations in the heart rate [28]. In addition, stroke volume variation is unreliable in patients who spontaneously breathe. Thus, this study considered only stroke volume as a parameter to evaluate the effects of fluid challenge. However, clinicians may benefit from this information under various conditions such as prolonged surgery, cesarean delivery complicated with profound bleeding, and cesarean delivery under general anesthesia with mechanical ventilation. Furthermore, continuous noninvasive arterial pressure monitoring reportedly detected more hypotension episodes [29], and the automated vasopressor infusion system based on these monitoring systems may help achieve a low incidence of postspinal hypotension during cesarean delivery [30].

This study had several limitations. First, we could not investigate the effect of GDFT preload on the reduction of phenylephrine dose for the treatment of postspinal hypotension. Despite phenylephrine being the first-line treatment in many institutions, it is not available in our country. However, growing evidence suggests that norepinephrine may be a noninferior or superior vasopressor compared with phenylephrine because of its lower risk of adversely affecting fetal acid–base status [31]. Second, no consensus exists regarding the GDFT protocol for the obstetric population. A dose of 4 mL/kg over 5 minutes of crystalloid reportedly reliably detects fluid responders and nonresponders [32]. In addition, a more rapid infusion of fluid volume increases the proportion of fluid responders [33]. We administered 3 mL/kg within 3 minutes based on actual body weight. This fluid dose is higher than the 4 mL/kg for ideal body weight, and the fluid challenge duration is shorter than 5 minutes. Therefore, we contend that this GDFT protocol efficiently maximizes the parturient's stroke volume before spinal anesthesia. Third, a higher iv fluid requirement for GDFT is expected for populations with higher weight than our cohort; the iv fluid requirement may be more prominently different between the GDFT and 1000 mL fixed-volume preload protocol; thus, our result may not be completely applicable to that population.

In conclusion, the CNAP-based GDFT preload protocol is not superior to a 1000 mL fixed-volume preload protocol in ameliorating postspinal hypotension during cesarean delivery, but GDFT may reduce postspinal nausea.

## Figures and Tables

**Table 1:** Patient characteristics.

	Control group (*N* = 34)	GDFT group (*N* = 37)	*p* value
Age (y)	35.6 (3.7)	36.6 (4.6)	*p* = 0.3376
Weight (kg)	67.4 (7.6)	69.6 (8.2)	*p* = 0.2526
Height (cm)	160 (5.7)	159.8 (4.7)	*p* = 0.9698
Gestation (wk)	38.1 (0.8)	37.5 (0.9)	*p* = 0.0109
Cesarean indication (*N*)			*p* = 0.2158
Previous cesarean delivery or myomectomy	21	17	
Breech presentation	4	7	
Cephalopelvic disproportion	3	1	
Placental previa	1	6	
Other	5	6	

Data are the mean (SD) or number.

**Table 2:** Intraoperative maternal profiles.

	Control group (*N* = 34)	GDFT group (*N* = 37)	*p* value
Fluid protocol time (min)	8.4 (3.5)	9.2 (3.5)	*p* = 0.3175
The end of fluid protocol to anesthesia time (min)	13.4 (5.9)	13.9 (8.0)	*p* = 0.7630
Total fluid (mL)	1132 (108)	1247 (202)	*p* = 0.0044
Spinal bupivacaine dose (mg)	11.2 (0.5)	11.2 (0.5)	*p* = 0.7035
Sensory blockade			*p* = 0.6456
*T*_6_ (*N*; %)	0 (0%)	1 (2.70%)	
*T*_5_ (*N*; %)	8 (23.53%)	9 (24.32%)	
*T*_4_ (*N*; %)	15 (44.12%)	11 (29.74%)	
*T*_3_ (*N*; %)	8 (23.53%)	12 (32.43%)	
*T*_2_ (*N*; %)	3 (8.82%)	4 (10.81%)	
Spinal to delivery time (min)	19.1 (4.3)	17.9 (6.9)	*p* = 0.3995
Postspinal hypotension (*N*; %)	27 (79.4%)	27 (73.0%)	*p* = 0.5864
Norepinephrine dose (*μ*g)	12.5 (10.6)	15.1 (12.8)	*p* = 0.3512
Maternal adverse effects (*N*; %)			
Nausea	21 (61.76%)	13 (35.14%)	*p* = 0.0332
Dizziness	11 (32.35%)	14 (37.84%)	*p* = 0.8040
Bradycardia	3 (8.82%)	5 (13.51%)	*p* = 0.7121
Shiver	3 (8.82%)	3 (8.11%)	*p* = 1.0000

SA = spinal anesthesia; data are mean (SD).

**Table 3:** Neonatal profiles.

	Control group (*N* = 34)	GDFT group (*N* = 37)	*p* value
Apgar score	9 (9-9 (8-9))	*N* = 37	
1 minute	9 (9-9 (9-9))	9 (9-9 (4-9))	*p* = 0.9331
5 minutes		9 (9-9 (8-9))	*p* = 0.3378
	Control group (*N* = 32)	GDFT group (*N* = 36)	*p* value
UV pH	7.36 (0.02)	7.35 (0.04)	*p* = 0.4778
UV pO_2_ (mmHg)	33.9 (6.7)	31.5 (7.6)	*p* = 0.1899
UV pCO_2_ (mmHg)	38.1 (4.0)	39.0 (4.6)	*p* = 0.4067
UV base excess	-4.3 (1.7)	-4.3 (1.8)	*p* = 0.9716
	Control group (*N* = 22)	GDFT group (*N* = 23)	*p* value
UA pH	7.34 (0.03)	7.32 (0.05)	*p* = 0.0734
UA pO_2_	28.2 (13.3)	23.3 (6.0)	*p* = 0.1149
UA pCO_2_	42.8 (5.1)	46.4 (6.5)	*p* = 0.0465
UA base excess	-3.0 (1.8)	-2.8 (2.2)	*p* = 0.6995

Values are the mean (SD) or median (IQR (range)); UV = umbilical vein; UA = umbilical artery.

## Data Availability

Data is available on request.
